# Development of a homogenous high-throughput assay for inositol hexakisphosphate kinase 1 activity

**DOI:** 10.1371/journal.pone.0188852

**Published:** 2017-11-29

**Authors:** Michael Wormald, Gangling Liao, Martha Kimos, James Barrow, Huijun Wei

**Affiliations:** 1 Department of Pharmacology and Molecular Sciences, Johns Hopkins University, Baltimore, Maryland, United States of America; 2 Drug Discovery Division, Lieber Institute for Brain Development, Baltimore, Maryland, United States of America; Pennsylvania State University, UNITED STATES

## Abstract

Inositol pyrophosphates have been implicated in a wide range of cellular processes. Inositol hexakisphosphate kinase 1 catalyzes the pyrophosphorylation of inositol hexakisphosphate into inositol 5-diphospho-1,2,3,4,6-pentakisphosphate which is important in numerous areas of cell physiology such as DNA repair and glucose homeostasis. Furthermore, inositol 5-diphospho-1,2,3,4,6-pentakisphosphate is implicated in the pathology of diabetes and other human diseases. As such there is a demonstrated need in the field for a robust chemical probe to better understand the role of inositol hexakisphosphate kinase 1 and inositol pyrophosphate in physiology and disease. To aid in this effort we developed a homogenous coupled bioluminescence assay for measuring inositol hexakisphosphate kinase 1 activity in a 384-well format (Z’ = 0.62±0.05). Using this assay we were able to confirm the activity of a known inositol hexakisphosphate kinase 1 inhibitor *N*2-(*m*-trifluorobenzyl), *N6*-(*p*-nitrobenzyl)purine. We also screened the Sigma library of pharmacologically active compounds at 10μM concentration and found 24 hits. Two of the most potent compounds were found to have an IC_50_ less than 5μM. The use of this high-throughput assay will accelerate the field towards the discovery of a potent inositol hexakisphosphate kinase 1 inhibitor.

## Introduction

Inositol phosphates have been recognized as important signaling molecules that are involved in a whole host of cellular processes ranging from growth to apoptosis [1–3]. Of these, inositol hexakisphosphate (IP6) can be pyrophosphorylated into a molecule called inositol 5-diphospho-1,2,3,4,6-pentakisphosphate (5PP-IP5) [4]. This compound contains a high-energy phosphoanhydride bond that allows it to participate in a different, but equally diverse, set of physiological processes than the inositol phosphates. Three inositol hexakisphosphate kinases (IP6K1, IP6K2 and IP6K3) have been found to catalyze the phosphorylation of IP6 to 5PP-IP5 and produce the vast majority of these molecules in the cell [4].

A number of IP6K genetic knockout experiments have been conducted in both *in vitro* and *in vivo* systems due to the wide range of processes that implicate 5PP-IP5. These studies have illuminated distinct phenotypic differences between the three family members. IP6K1, for example, is involved in glucose and insulin homeostasis, DNA repair, and chromatin modifications [5–9]. IP6K2 is important in promoting cell death [10]. Expression of IP6K3 is primarily restricted to the brain and as such there is evidence it is involved in neuronal formation [11]. Recently, IP6K3 has also been shown to play an important role in metabolic regulation [12].

In a diet induced obesity (DIO) model of diabetes, IP6K1 knock-out mice were resistant to weight gain and performed better in insulin and glucose tolerance tests when compared to their wild-type counterparts suggesting the protein’s contribution to a diabetic phenotype [9]. The interpretation of the knock-out model is complicated by the fact that IP6K1 possesses a non-enzymatic stimulatory effect on glycogen synthase kinase 3 beta (GSK3β) activity via a protein-protein interaction [6]. To this end, an IP6K1 catalytic inhibitor may help delineate the importance of the enzyme’s kinetic activity from its protein-protein interactions as they relate to inositol pyrophosphate physiology, specifically metabolic regulation. Currently only one small molecule inhibitor of IP6K1 is known. *N*2-(*m*-trifluorobenzyl), *N6*-(*p*-nitrobenzyl)purine (TNP) was discovered in a screen as a weak inhibitor of IP3K and later found to more potently inhibit IP6K1 [13]. TNP was an experimentally useful chemical complement to the genetic IP6K1 knock-out mice in a DIO model [14]. Like the knock-out animals, those mice treated with TNP were found to show better performance in insulin and glucose tolerance tests [14]. However, TNP is not an ideal tool compound for studying inositol pyrophosphate physiology due to its poor pharmacokinetic properties [14], insolubility in aqueous media, and pan-IP6K inhibitory profile [13]. Previous work with IP6K1 has shown it to be involved in regulation of neuronal migration [15]. However, TNP has not been able to be used to study this effect in the brain or in behavioral studies due to its low brain penetration. To compound these issues, current assays used to measure IP6K1 activity utilize HPLC separation of the inositol phosphates followed by radioactive or metal-dye detection of the fractionated molecules. Both of these assays require extensive run times and expensive equipment [16]. Therefore, a new assay technology to measure IP6K1 activity is needed in order to develop a less expensive, less time-consuming, higher throughput screening assay for inhibitors against IP6K1.

The IP6K family has an unusually low affinity for ATP, when compared to protein kinases, with a K_m_ value reported to be roughly 1mM [17,18]. Traditional kinase detection technologies such as P^33^ based scintillation proximity assays, antibody base detection of ADP by HTRF, or ATP coupled luciferase assays are challenging to implement with high ATP concentrations. The Promega ADP-Glo Max assay kit is able to detect ADP production in the presence of up to 5mM ATP and the ADP-Glo assay has been used in an HTS assay for another phosphoinositol kinase—PI5P4K [19]. In this study we have developed an HTS assay for IP6K1 using the Promega ADP-Glo Max kit and confirmed the inhibitory effect of TNP. We have validated the assay’s ability to perform in a high-throughput manner by screening the Sigma library of pharmacologically active compounds (LOPAC). Two potent hits identified from this screen were further confirmed by dose-titration demonstrating that use of this assay technology is suitable for the discovery of novel IP6K1 inhibitors.

## Materials and methods

### IP6K1/2/3 purification

Human IP6K1 (NCBI accession# Q92551), human IP6K2 (NCBI accession# Q9UHH9), and human IP6K3 (NCBI accession# Q96PC2) were purified from C43 *E*. *coli* transformed with pGEX-6P-2 expression vector containing GST tagged IP6K1, IP6K2, or IP6K3. The bacteria were grown overnight in a starter culture containing LB (Lennox) and 100μg/mL ampicillin (Sigma). The starter culture was diluted 1:100 into LB (Lennox) and allowed to grow to an optical density of 0.8 at 37°C when protein expression was induced with 1mM IPTG (Calbiochem) for 5hrs. The cells were pelleted with 3000RCF at 4°C and frozen at -80°C. Later, the bacteria was re-suspended in lysis buffer (20mM Tris, 200mM NaCl, 5mM DTT, 1% Triton-X, 0.5% IPEGAL, 1mg/mL lysozyme, and 1 protease inhibitor tablet (Sigma) per 100mL buffer, pH 7.4) and rotated on an end over end shaker for 30 min at 4°C followed by sonication. Cellular debris were pelleted via centrifugation (18,000 RPM, JA-20 Rotor, 30mins, 4°C) and the soluble lysate was added to Glutathione Sepharose 4B resin (GE Life Sciences) and rotated on an end over end shaker at 4°C for 2hrs to allow for binding. The resin bound protein was isolated via batch purification and washed with 5x10mL lysis buffer without protease inhibitor or lysozyme. The resin was then treated with PreScission Protease (GE Life Sciences) to cleave the GST tag and allow the release of IP6K1/2/3 from the resin. After 16hrs of Prescission Protease treatment, the resin was spun down, the purified protein was eluted, and dialyzed into a solution containing 50mM Tris, 50mM NaCl, 1mM DTT, and 20% glycerol. The kinase was aliquoted and stored at -80°C.

### IP6K1 kinetic and activity assays

IP6K1 activity was measured using the ADP-Glo Max kit (Promega). Assays were carried out in Corning® low volume 384 well white flat bottom polystyrene NBS™ microplates with a final volume of 5μL containing 30–120nM IP6K1 and 6.25–500μM IP6 (Sigma) in kinase buffer (50mM Tris, 10mM MgCl_2_, 2.5mM DTT, pH 6.9). Reactions were performed in a 37°C incubator and started with the addition of ATP to a final concentration of 62.5–2000μM; time course reactions were performed in a hot water bath at 37°C. Reactions were quenched with 5μL of Promega ADP-Glo reagent to deplete the remaining ATP at the corresponding time points and incubated for 1hr at room temperature in the dark. Then, 10μL of Promega Kinase Detection reagent was added and incubated for 1hr at room temperature in the dark to convert ADP generated by IP6K1 into ATP, which is then utilized in a luciferase/luciferin reaction to generate a bioluminescent signal. Luminescence was detected with a Tecan Infinite M100 Pro plate reader. The amount of ADP produced by IP6K1 was determined by correlating the observed luminescence to a luminescence signal standard curve generated from various concentrations of ADP. A new standard curve was generated for each concentration of ATP tested such that the total amount of nucleotides (ATP+ADP) at every point on the curve was equal to the concentration of ATP used in the experiment. Standard curves typically began with 100μM or 50μM ADP and were serially 2-fold diluted 8 times. We found that the presence of IP6 has no effect on ADP measurement and therefore did not include IP6 in our ADP standard curve measurement (S1 Appendix). Kinetic determinations were carried out at 15 minutes in the linear phase of the enzymatic reaction with <10% substrate turnover using 15ng IP6K1. Nonlinear regressions and curve fitting to the Michaelis-Menten equation were performed in GraphPad Prism 7.

### IP6K inhibition assays

For the dose response assays, 7–10 serial 3-fold compound dilutions were prepared in DMSO and then each compound was diluted with assay buffer (50mM Tris, 10mM MgCl_2_, 2.5mM DTT, 0.02% Triton X-100, pH 6.9) to reach the desired inhibitor concentrations in 25% DMSO. Then, 1μL of each dilution was added to a Corning® low volume 384 well white flat bottom polystyrene NBS™ microplate. A ‘kinase master mix’ that contained IP6K1, IP6, and ATP was prepared on ice, mixed gently, and 4μL of this was added to each compound resulting in a total reaction volume of 5μL with 5% DMSO. The final concentrations for IP6K1, IP6, and ATP were 60nM, 100μM, and 1mM respectively. The reaction plate was quickly mixed on a plate shaker and centrifuged for 10 seconds at 1500x*g*. The plate was then mixed and centrifuged a second time. The mixed reaction plate was placed into a 37°C incubator and rotated on an orbital shaker at 800rpm for 30min. The reaction was terminated with 5μL of Promega ADP-Glo reagent for 1hr followed by 10μL Promega Kinase Detection reagent for 1hr at room temperature in the dark. Luminescence generated was read on a Tecan Infinite M100 Pro plate reader. Samples were run in quadruplicate and the data was analyzed using a four-point variable slope inhibition parameter on GraphPad Prism 7.

The effect of Myricetin and 6-Hydroxy-DL-Dopa on IP6K2 and IP6K3 were tested as described above for IP6K1 with some variations. The IP6K2 assays used 7.5nM of enzyme and the IP6K3 assays used 120nM of enzyme with a 2-hour reaction time.

### IP6K1 high-throughput screen

The Sigma LOPAC was obtained as 10mM DMSO stock solutions from the Johns Hopkins ChemCore facility. Each compound was first diluted to 50μM and 25% DMSO in assay buffer. The inhibition assay was performed as described above with 1μL of compound and 4μL of kinase master mix so that the final compound concentration was 10μM in 5% DMSO. Myricetin at 100μM was used as the 100% inhibition control. Assay robustness was estimated using the Z’ factor, Z’ = 1-3(σ_positive_+σ_negative_)/|μ_positive_-μ_negative_|, where the positive controls are treated with DMSO and the negative controls are treated with 100μM Myricetin. Hits were determined based by the Z score, which is calculated as Z = (X_i_-X_avg_)/S_x_, where X_i_ is the raw measurement on the i^th^ compound, X_avg_ and S_x_ are the mean and the standard deviation, respectively, of all measurements within the plate [20]. Hits identified from the screen were repeated in the assay in quadruplicate for confirmation. These confirmed hits were then tested to determine if they inhibited the enzymes involved in the detection steps of the kit. Possible interference of the compounds was assessed by measuring the degree to which they inhibited the signal produced by a 25μM ADP in the presence of 975μM ATP. This experiment was run as described above for the inhibition assays in the absence of enzyme and IP6.

### Inositol polyphosphate separation by PAGE

IP6 and 5PP-IP5 were separated by high concentration PAGE as described previously [21]. Briefly, 33.3% polyacrylamide gels were cast and pre-run for 20–60 mins at 300V and 4°C in TBE. IP6K1 enzymatic reactions were carried out in assay buffer (50mM Tris, 10mM MgCl_2_, 2.5mM DTT, pH 6.9) with 1mM ATP, 250μM IP6, and 60nM IP6K1 for 2 hours at 37°C in an Eppendorf micro-centrifuge tube and stopped with 2μL 500mM EDTA. Reactions with Myricetin, 6-Hydroxy-DL-Dopa, or TNP present were run with 10μM compound present. The gel was loaded with 50μL of, 1mM IP6, 1mM ATP, and 1mM in-house synthesized 5PP-IP5 as standards. Standards were made in assay buffer with 2μL EDTA added. Additionally, 100μL of the control and inhibition reactions were loaded onto the gel to ensure enough analyte was present to visualize with Toluidine Blue. Gels were run overnight at 300V and 4°C until the Orange G dye front was 10cm from the bottom of the plate at which point the run was stopped and the gel stained for 2min with a Toluidine Blue staining solution (20% methanol; 2% glycerol; 0.05% Toulidine Blue) and destained for 5–10 minutes with tap water. The gel was then visualized using a Syngene PXi gel-imaging system. The contrast and brightness was adjusted to be able to clearly see demarcations between the analytes and the gel background.

## Results and discussion

### Design and optimization of the IP6K1 assay

**Fig 1:**
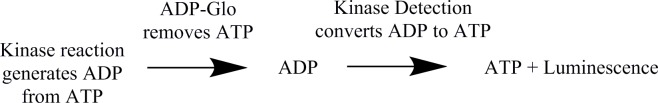
Promega ADP-Glo Max assay workflow. IP6K1 was incubated with ATP and IP6 to generate ADP and PP-IP5. The excess ATP in the reaction was removed with the ADP-Glo reagent leaving the ADP concentration unchanged. The Kinase Detection reagent converts ADP to ATP, which can then react with luciferase to generate a bioluminescent signal that is directly proportional to IP6K1 activity.

**Fig 2:**
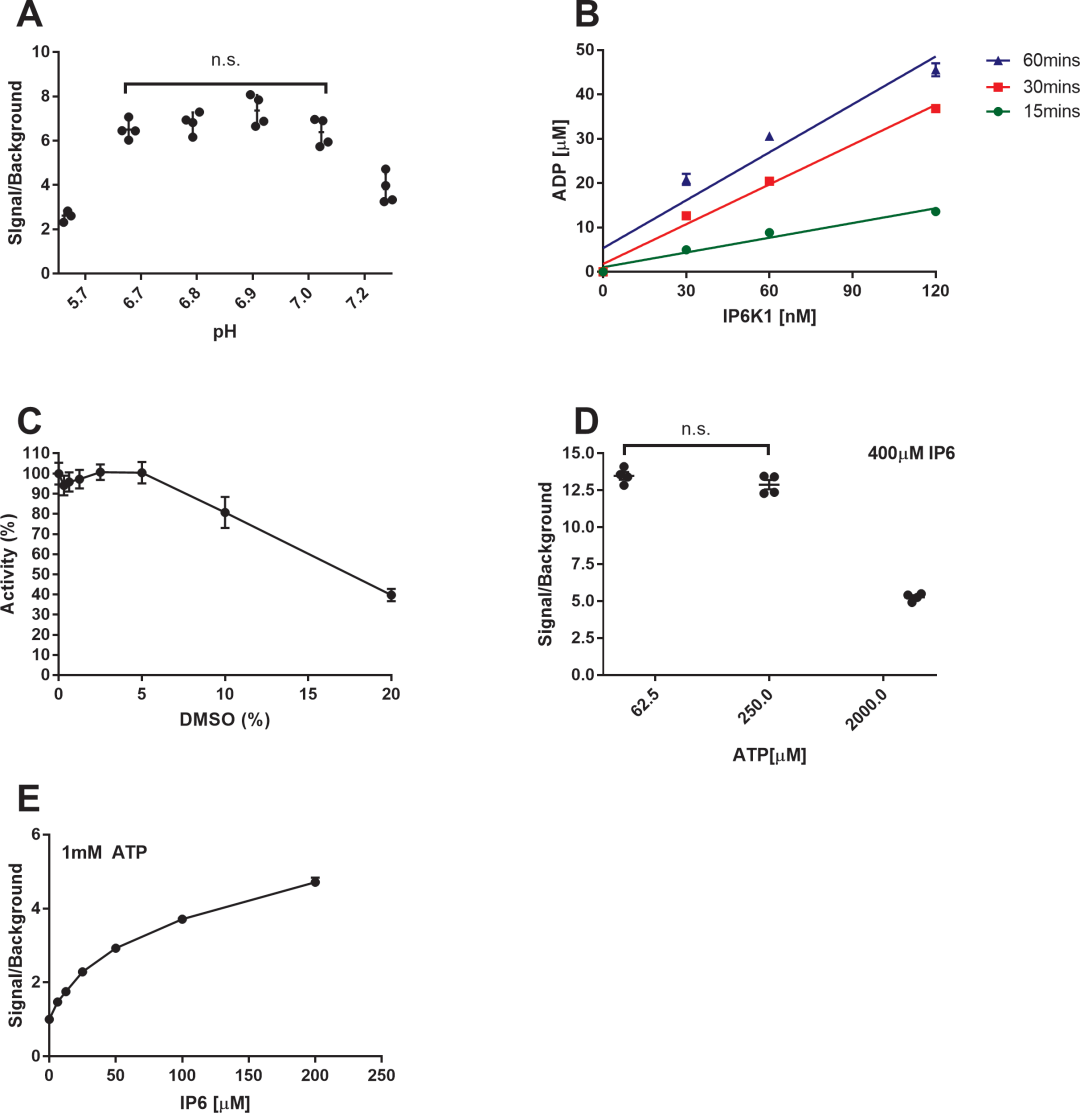
Optimization parameters of the IP6K1 high throughput assay. Various assay parameters were investigated and optimized. (A) Buffer pH was explored using buffers with 6 different pH values between 5.7 and 7.2 units, 1mM ATP, 500μM IP6, and 120nM IP6K1 for 30 minutes at 37°C. (B) Three IP6K1 concentrations were tested at three time points with 300μM ATP and 250μM IP6 to determine conditions that gave good signal and displayed linearity. (C) The percentage of DMSO tolerated by the assay was measured with 0.3125–20% DMSO, 1mM ATP, 500μM IP6, and 120nM IP6K1 for 30 minutes at 37°C. Concentrations higher than 5% were detrimental to assay performance. (D) ATP concentrations of 62.μM, 250μM, and 2000μM were tested with 400μM IP6 and 60nM IP6K1 for 30 minutes at 37°C. (E) IP6 concentrations of 6.25–200μM were tested with 1mM ATP and 60nM IP6K1 for 30 minutes at 37°C. The signal/background ratio is defined as the luminescent signal of the reaction divided by the signal generated from the same reaction in the absence of IP6. ADP concentrations are calculated by correlating the luminescence produced by a known standard of ADP to that of the experimental condition. Percent activity is defined as 100*(μ_experimental_–μ_negative_)/(μ_positive_–μ_negative_) where positive and negative controls are the experiment with and without IP6 present. All data points are replicated in quadruplicate and represented as the mean ± SEM. Non-significance was determined via one-way ANOVA testing where p > 0.05.

The IP6K1 enzyme is reported to have a K_m_ for ATP in the range of several hundred micromolar to low millimolar [17,18], therefore it is essential that the assay technology that detects nucleotides must be exquisitely sensitive to small amounts of ADP in a large excess of ATP. To accomplish this goal we utilized the Promega ADP-Glo Max Assay to optimize a high-throughput method of detecting IP6K1 activity (Fig 1). The assay is carried out in three steps: 1) the kinase reaction where ADP is generated from ATP, 2) the depletion reaction where residual ATP is removed from the mixture, and 3) the regeneration and detection reactions where ADP is converted back to ATP which is transformed to a luminescence signal via a coupled reaction with luciferase and luciferin. As a result the amount of luminescence produced is directly proportional to the amount of ADP produced by IP6K1. Various assay conditions were tested including buffer pH (Fig 2A), reaction time and kinase concentration (Fig 2B), DMSO sensitivity (Fig 2C), ATP concentration (Fig 2D), and IP6 concentration (Fig 2E).

We initially explored the effect of pH on the catalytic activity of IP6K1. We found that IP6K1 is very sensitive to small changes in buffer pH (Fig 2A). The enzyme preformed robustly when pH was slightly acidic between 6.7 and 7.0 units; however, values below 6 or above 7 began to markedly impair enzyme activity. We chose pH 6.9 to carry out further experiments.

Next we examined the activity of different enzyme concentrations at different time points to determine assay linearity (Fig 2B). Reactions run for 15 and 30 minutes showed good linearity compared to the 60-minute condition. The 30-minute time point with 60nM of IP6K1 was chosen for subsequent experiments due to the observations that the enzyme is stable and displays linear kinetics at 30 minutes, the increased ADP production allows for a larger signal window compared to the 15 minute time point, and 60nM IP6K1 allows us to conserve enzymatic reagent while still remaining somewhat sensitive to highly potent inhibitors.

The sensitivity of the assay to DMSO was determined by running the reaction with a total DMSO concentration of up to 20%. It was discovered that DMSO is well tolerated by this assay up to 5% total concentration (Fig 2C).

One of the major sources of background signal in this assay comes from the high amounts of ATP used. To this end we determined the effect that ATP concentration had on the signal/background ratio (Fig 2D). Here we are presenting the ratio of the signal produced by the IP6K1 catalyzed phosphorylation of IP6 over the same reaction without IP6 at 62.5μM, 250μM, and 2000μM ATP. Unsurprisingly, the reactions with lower ATP concentrations had a larger signal/background ratio due to the lower background, despite a smaller absolute signal. Although the 62.5μM and 250μM ATP conditions had statistically insignificant signal/background ratios we chose 62.5μM ATP for our initial screening efforts due to the lower background observed in that experiment.

Similarly, the assay signal/background ratio was determined at various IP6 concentrations (Fig 2E). The ratio changed from 3.7 at 100μM IP6 to 4.7 at 200μM IP6. At 100μM IP6 the curve appears to be reaching a plateau where doubling the IP6 concentration beyond 100μM results in just over a 25% increase in the signal/background ratio. IP6 is already saturating IP6K1at 100μM and it was determined that further saturation would not yield significant increases with regards to the signal/background ratio and, indeed, might heavily bias screening efforts against IP6 competitive inhibitors.

As a result of these optimization experiments it was determined that best experimental conditions with which to begin our high-throughput screening efforts were 62.5μM ATP, 100μM IP6, 60nM IP6K1 in 25mM Tris-HCl (pH 6.95), 10mM MgCl_2_ and 2.5mM DTT at 37°C for 30min with less than 5% final DMSO concentration.

### Determining the K_m_ and V_max_ for ATP

**Fig 3:**
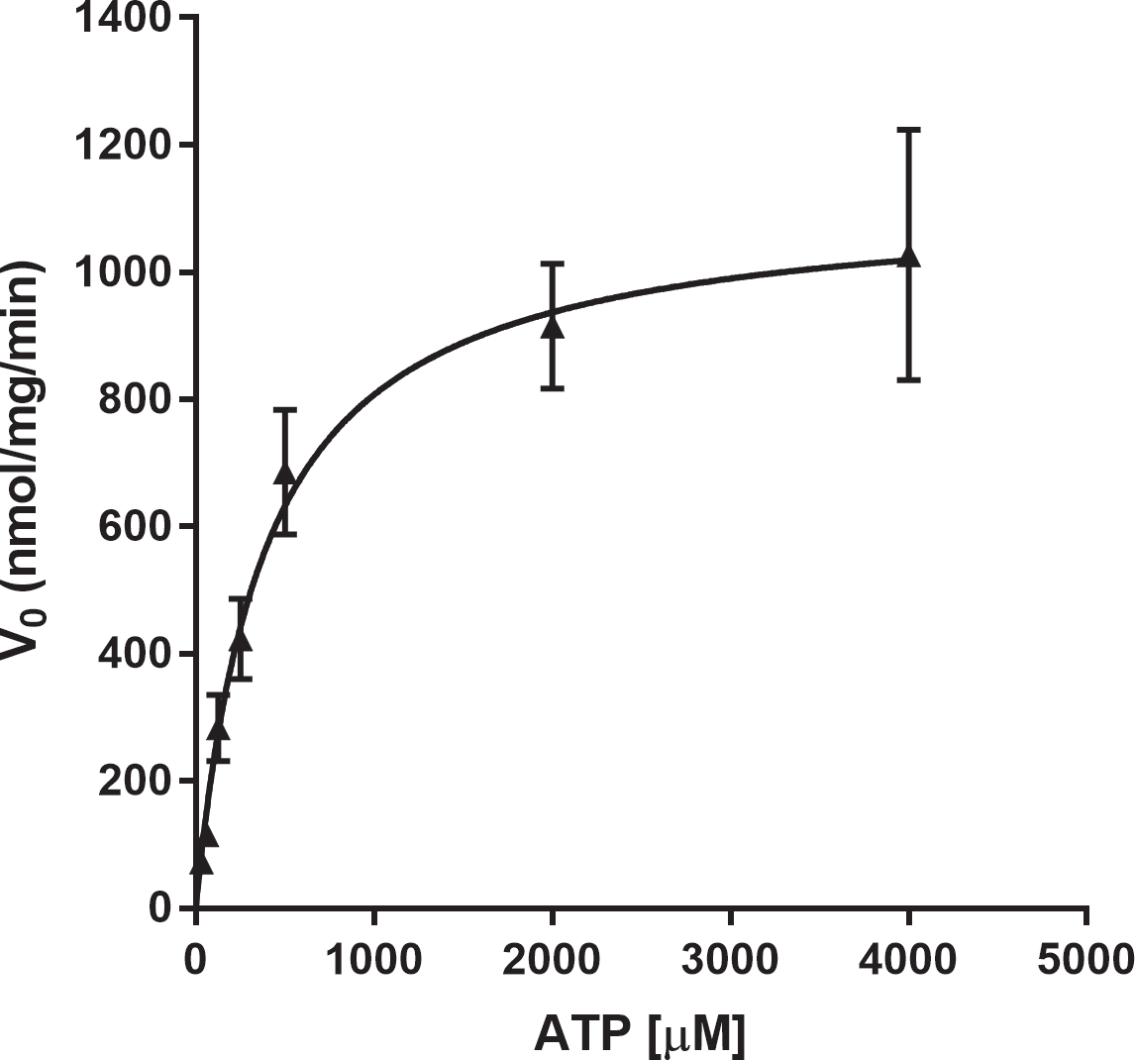
IP6K1 enzyme kinetics. Michaelis-Menten kinetic parameters were determined for IP6K1 at various concentrations of ATP. The K_m_ and V_max_ parameters for ATP were determined to be 382±44μM and 1116±41nmol/min/mg respectively. The experiment was conducted with 400μM IP6 with variable amounts of ATP and 15ng IP6K1 for 15 minutes. All data points (n = 4–6) are represented as the mean ± SEM and fit to the Michaelis-Menten equation for analysis.

The K_m_ of ATP for IP6K1 was reported previously to be 1mM by quantifying the production of ^3^H-PP-IP5 using ^3^H-IP6 as the substrate [18]. To determine the K_m_ of ATP using our ADP-Glo Max assay, the kinase reaction was carried out with 400μM of IP6 and ATP concentrations ranging from 31.25μM to 2mM. The ADP production at 15mins was measured. The initial reaction velocity (V_0_) was calculated and plotted with each ATP condition. GraphPad Prism 7 was used to run the non-linear regression and fit the data to the Michaelis-Menten equation. The results showed that K_m_ = 382±44μM and V_max_ = 1116±41nmol/min/mg (Fig 3). The Michaelis-Menten constants when IP6 is the varied substrate were unable to be determined. The low concentration of IP6 used in these experiments leads to small amounts of 5PP-IP5 and ADP production that fall outside of the assay’s detection limit in the presence of saturating ATP.

### Assay validation with TNP

**Fig 4:**
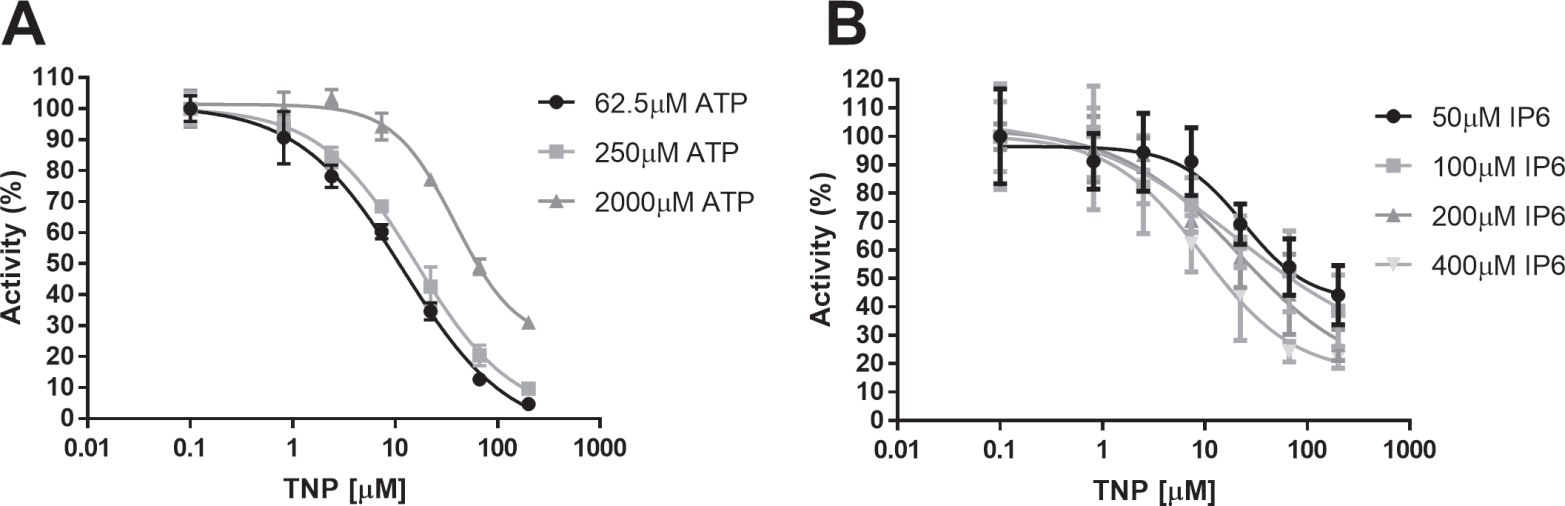
Dose response inhibition of IP6K1 by TNP in the presence of different concentrations of ATP and IP6. TNP was confirmed to compete with ATP by binding to IP6K1. (A) IC_50_ values increased from 12±1.1μM to 39±1.1μM when ATP concentrations increased from 62.5μM to 2000μM indicating competitive inhibition. The reaction was run with 400μM IP6 and 60nM IP6K1 for 30 minutes at 37°C. (B) IC_50_ values decreased slightly from 24±1.5μM to 9.5±1.5μM as IP6 concentrations were increased from 50μM to 400μM. Reactions were carried out with 300μM ATP and 60nM IP6K1 for 15 minutes. Percent activity is defined as 100*(μ_experimental_-μ_negative_)/(μ_positive_–μ_negative_) where positive is the experimental result with no inhibitor present and negative is the signal produced by the reaction with no IP6 present. All data points are replicated in quadruplicate and represented as the mean ± SEM.

TNP is a purine analog that has been shown to have IP6K1 inhibitory properties [13]. We confirmed the activity of TNP as a relatively weak inhibitor of IP6K1 with IC_50_ = 12±1.1μM when the assay was performed with 62.5μM ATP. In addition, IC_50_ values of TNP increased to 16±1.1μM and 39±1.1μM with 250μM and 2mM ATP present suggesting that TNP is an ATP competitive inhibitor (Fig 4A).

IP6 is not competitive with TNP. Instead, increasing IP6 concentration shows a slight 2 fold increase in potency over a range of IP6 concentrations (Fig 4B). As concentrations were increased to 50μM, 100μM, 200μM and 400μM the IC_50_ values of TNP changed to 24±1.5μM, 20±4.5μM, 21±1.8μM, 9.5±1.5μM respectively.

The potency of TNP in this assay is substantially lower than reported elsewhere [13] most likely due to our two-step compound dilution scheme. We are limiting the efficacy of very hydrophobic molecules, such as TNP, by diluting the 10mM compound DMSO stock first into a 25% DMSO/75% buffer solution. As a result, these types of compounds tend to nucleate and fall out of solution leading to an artificially low concentration of the molecule in the reaction. Access to a liquid handler with the capacity to dispense nanoliter and picoliter amounts of DMSO stock solution directly onto the assay plate would likely eliminate these dilution issues.

### Screening the LOPAC

The Sigma LOPAC is an annotated set of 1280 compounds with known activity often used to validate the performance of a high-throughput assay [22]. We initially screened the LOPAC against IP6K1 at single point 100μM compound concentration and 62.5μM ATP, which is much lower than the K_m_ value of ATP for IP6K1. Under these assay conditions, a signal/background ratio of 10.6 and Z’ of 0.79 were observed. However, it generated an unexpectedly high hit rate (>8%) presumably due to an over representation of ATP competitive inhibitors and nonspecific compound aggregation. Nevertheless, a few compounds, such as Myricetin, were able to inhibit 100% of the enzyme activity and performed much better than TNP.

**Fig 5:**
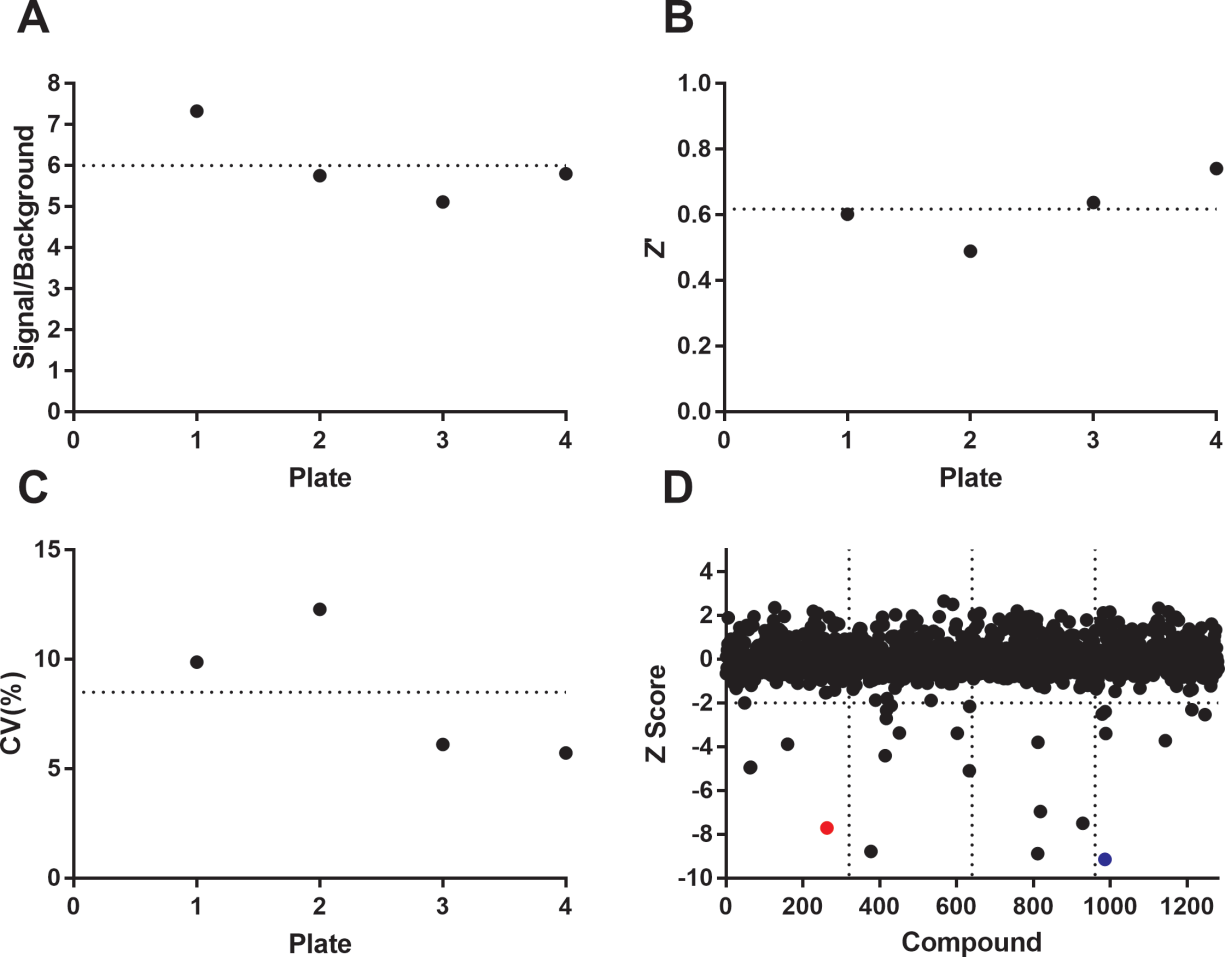
Assay validation and LOPAC screening results. The LOPAC was screened to assess the degree to which Promega’s ADP-Glo Max assay can be used to measure IP6K1 activity in a high-throughput manner. (A) Signal to background ratio represents the DMSO control divided by 100μM Myricetin inhibition control. Signal/background = 6.00±0.47. (B) Z’ factor = 0.62±0.05. (C) CV = 8.50±1.57%. (D) Plot of Z score for LOPAC compounds. Compounds below Z of -2.0 are considered hits. Myricetin is highlighted in red and 6-Hydroxy-DL-Dopa in blue. Data presented here are representative of a single LOPAC screen where each compound is screened at one 10μM concentration.

To lower the hit rate and deprioritize the ATP competitive inhibitors for IP6K1, we rescreened the LOPAC at 10μM compound concentration in the presence of 1mM ATP. Although ATP levels fluctuate drastically due to cell type and point in the cell cycle, a value of 1mM ATP is physiologically relevant as most cells maintain ATP concentrations between 1-5mM [23–26]. In order to alleviate false positives that may have resulted from compound aggregation, Triton X-100 was added to the assay buffer at 0.02% final concentration. Myricetin at 100μM was used as a control for complete inhibition. Higher levels of ATP can lead to increased residual ATP levels after the ATP depletion step, resulting in an increase in the signal background. As expected, the ratio of total signal over the signal with 100μM of Myricetin was reduced at 6.00 under these new conditions (Fig 5A). This assay also display a Z’ factor of 0.62±0.05 (Fig 5B) and a CV of 8.50±1.57% (Fig 5C), therefore, it is still robust enough for high-throughput screening. In total, 24 compounds were identified from this screen with Z score less than -2.0 representing a hit rate of 1.88% (Fig 5D). A full list of the LOPAC screened against IP6K1 and their Z scores are reported in a supplemental file (S2 Appendix).

### Validating LOPAC hits

**Fig 6:**
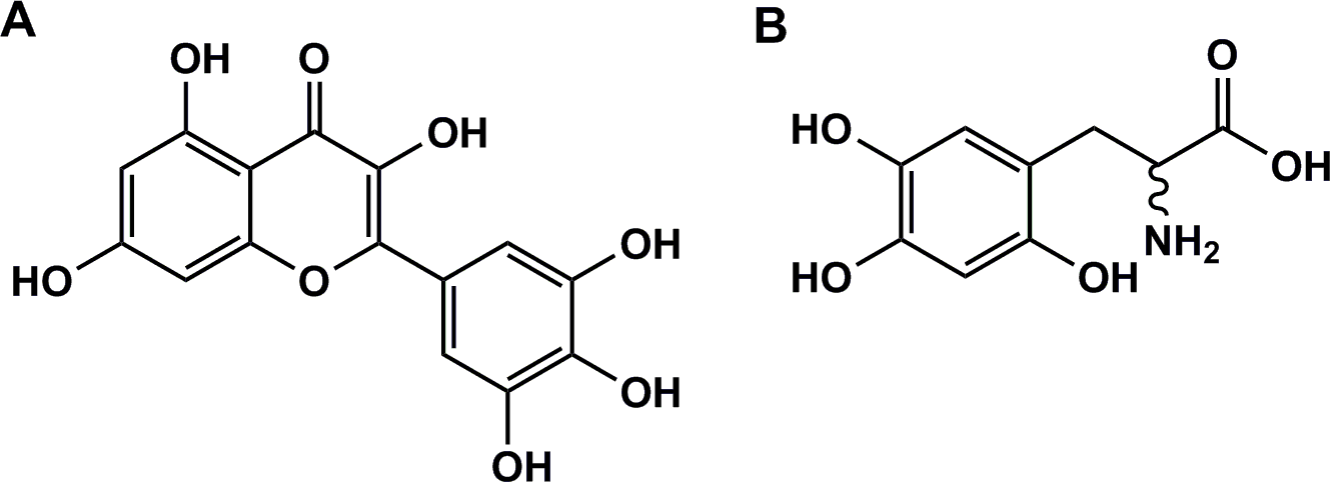
Chemical structure of inhibitors found in LOPAC screen. (A) Myricetin and (B) 6-Hydroxy-DL-Dopa.

**Fig 7:**
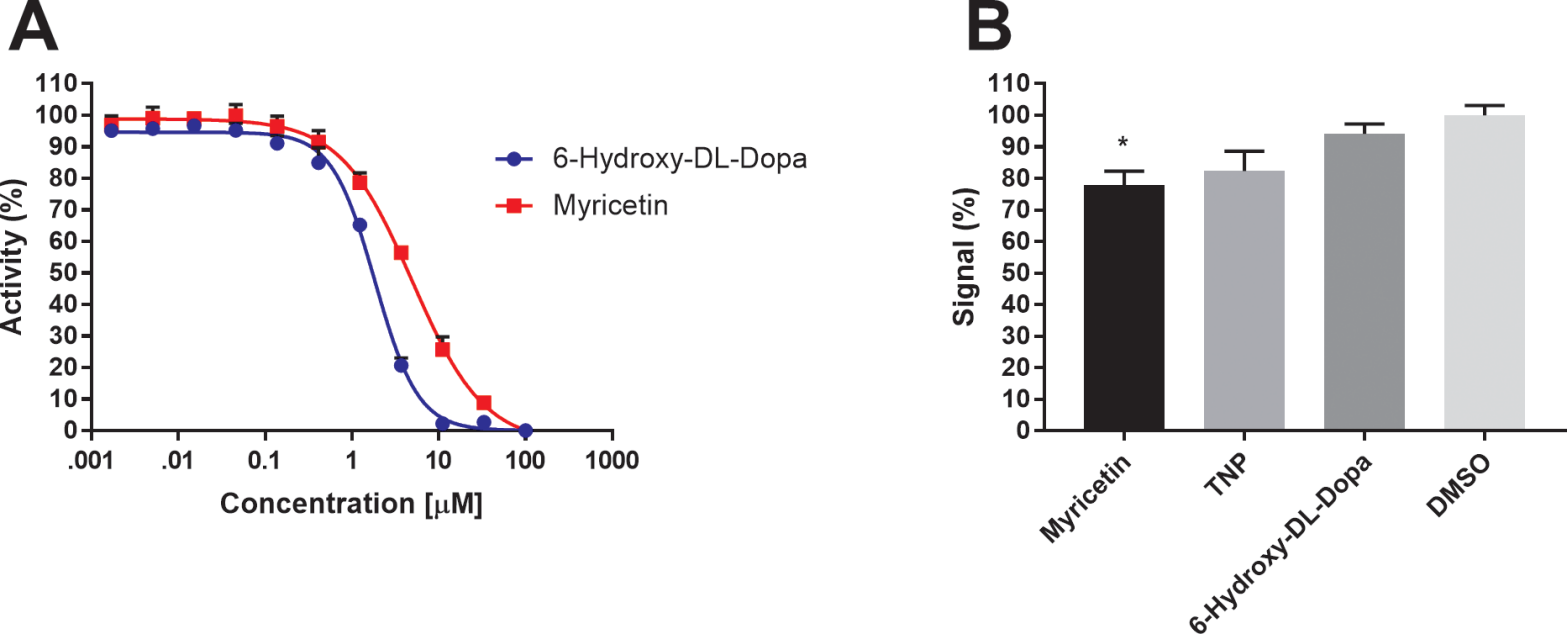
LOPAC hit validation and counter-screening. (A) Dose response assay for inhibition of IP6K1 by 6-Hydroxy-DL-Dopa and Myricetin. Reactions were run with 1mM ATP, 100μM IP6, 60nM IP6K1, for 30 minutes at 37°C. Myricetin and 6-Hydroxy-DL-Dopa were able to inhibit IP6K1 with an IC_50_ of 4.96±1.06μM and 1.84±1.03μM respectively. Percent activity is defined as 100*(μ_experimental_-μ_negative_)/(μ_positive_–μ_negative_) where positive is the experimental result with no inhibitor present and negative is the signal produced at the highest concentration of inhibitor. (B) IP6K1 inhibitors Myricetin, TNP, and 6-Hydroxy-DL-Dopa were tested at 10μM against 25μM ADP and 975μM ATP. Percent signal is defined as 100*(μ_experimental_–μ_negative_)/(μ_positive_–μ_negative_) where the positive is the 5% DMSO control and the negative contains no IP6. All data points are replicated in quadruplicate and represented as the mean ± SEM. Significance was determined via one-way ANOVA testing where p < 0.05.

All of the 24 hits from the LOPAC screen were polysulfonylated sodium salts, polyphenolic alkaloids, or contained one or more catechol moieties. The majority of these were dismissed from further analysis because they were thought to be unable to cross the cell membrane, contained motifs notoriously present in pan-assay interference compounds (PAINS) [27], or were too large for further chemical development. Two of the most potent compounds, Myricetin and 6-Hydroxy-DL-Dopa were selected for further analysis and validation, despite their ‘PAINS’ motifs, due to their small size and predicted ability to pass through a cell membrane (Fig 6). A 10-point dose-response curve was generated for each of these compounds against IP6K1. Myricetin and 6-Hydroxy-DL-Dopa were able to inhibit IP6K1 with an IC_50_ of 4.96±1.06μM and 1.84±1.03μM respectively (Fig 7A). When tested at 10μM against the detection steps in a counter-screen neither TNP nor 6-Hydroxy-DL-Dopa showed significant signal loss indicating that the vast majority of inhibition was via IP6K1 (Fig 7B). Myricetin was found to significantly inhibit the secondary reagents; however, Myricetin is drastically more potent when IP6K1 is included in the reaction.

### Myricetin and 6-Hydroxy-DL-Dopa directly inhibit IP6K1 production of 5PP-IP5

To further confirm Myricetin and 6-Hydroxy-DL-Dopa as bona fide IP6K1 inhibitors we conducted high concentration polyacrylamide gel electrophoresis experiments that have previously been shown to detect and separate IP6 from 5PP-IP5 generated by IP6K1 [21]. We incubated 60nM of IP6K1 with 250μM IP6, 1mM ATP, and 10μM of Myricetin, 6-Hydroxy-DL-Dopa, or TNP for 2 hours at 37°C. A higher concentration of IP6 and longer reaction time were used in these reactions when compared to the ADP-Glo Max assay in order to increase the amount of inositol phosphates present in the gel and achieve stronger staining. Reactions were quenched with 2uL 500mM EDTA and samples were run on a 33% polyacrylamide gel at 300V overnight. The following day the voltage was turned off when the Orange G dye front was roughly 10cm from the bottom of the plate and the gel was stained with Toluidine Blue. After a quick destaining process in tap water the gel was imaged in a Syngene PXi gel-imaging system. The destaining procedure was difficult to optimize. If the gel was destained for too short of a time then the background blue level would be excessively high and the bands could not be identified. If the destaining time was too long then the stained inositol phosphates would lose their dye at the same rate as the polyacrylamide gel, resulting in a very weak signal. The best results were obtained with roughly 2 minutes of staining followed by 5–10 minutes of destaining in tap water. These conditions produced visible blue bands noticeably above background.

**Fig 8:**
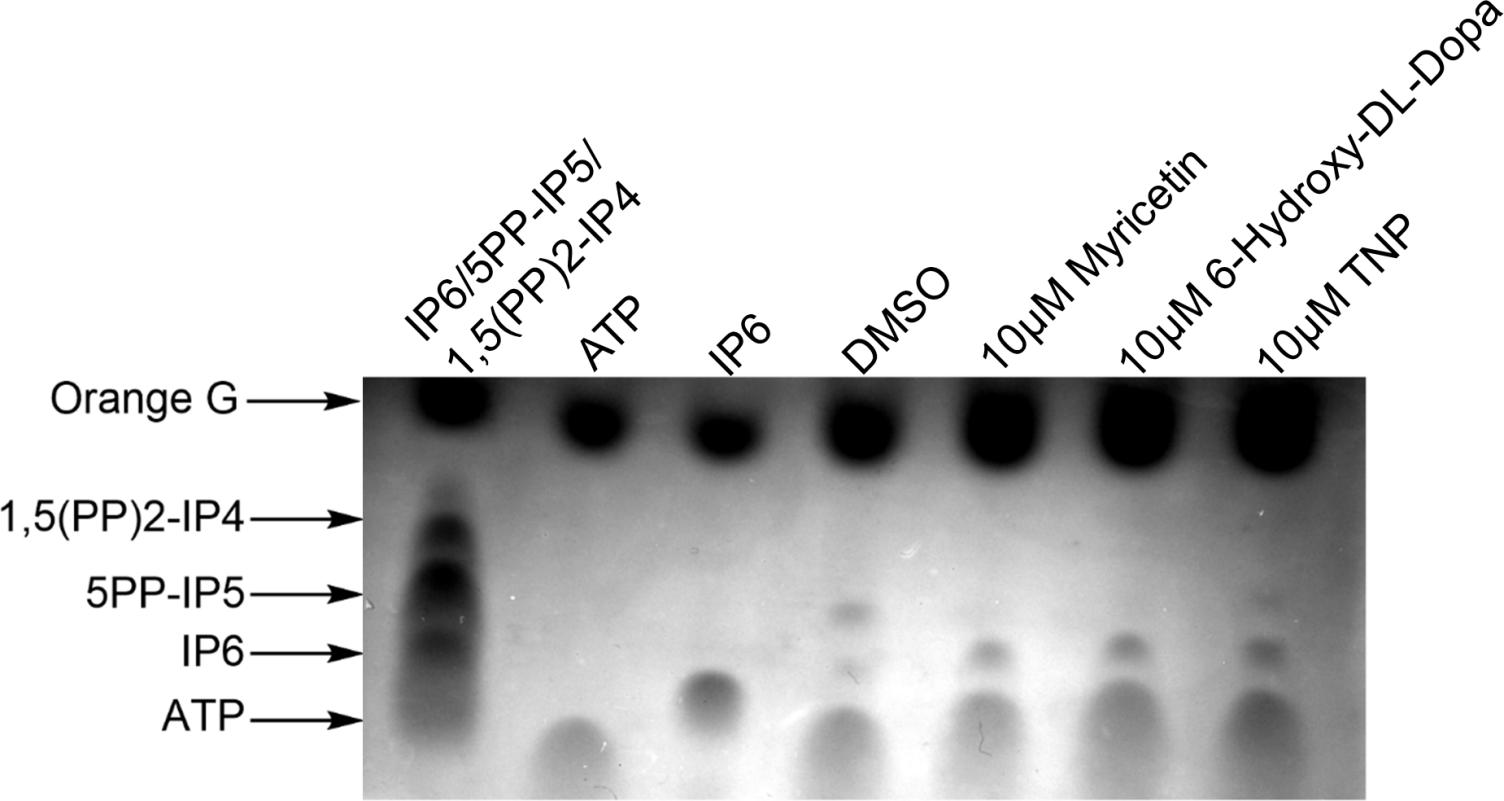
PAGE separation of IP6 and 5PP-IP5 shows Myricetin and 6-Hydroxy-DL-Dopa inhibit IP6K1 catalytic activity. A representative result of numerous gel separations is shown here. When incubated with Myricetin or 6-Hydroxy-DL-Dopa 5PP-IP5 production by IP6K1 is dramatically reduced. Each reaction is carried out with 10μM inhibitor, 1mM ATP, and 250μM IP6 with 60nM IP6K1 for 2 hours at 37°C.

The control reaction without any inhibitors shows that IP6K1 is able to produce 5PP-IP5 from IP6. This 5PP-IP5 production is inhibited below the limit of detection on the gel when 10μM of Myricetin or 6-Hydroxy-DL-Dopa are added to the reaction. TNP is also shown to inhibit 5PP-IP5 production in this experiment at 10μM albeit to a lesser degree than Myricetin or 6-Hydroxy-DL-Dopa as evidenced by the faint staining where 5PP-IP5 is observed (Fig 8).

This experiment confirms Myricetin and 6-Hydroxy-DL-Dopa as genuine inhibitors of IP6 pyro-phosphorylation catalyzed by IP6K1.

### Myricetin and 6-Hydroxy-DL-Dopa inhibit IP6K2 and IP6K3 activity

**Fig 9:**
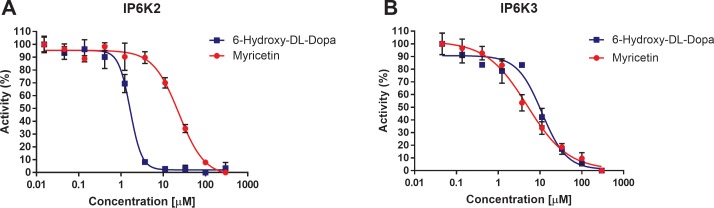
LOPAC hits inhibit IP6K2 and IP6K3. Dose response assay for inhibition of (A) IP6K2 and (B) IP6K3 by Myricetin and 6 -Hydroxy-DL-Dopa. Myricetin and 6-Hydroxy-DL-Dopa were able to inhibit IP6K2 with an IC_50_ of 23.41±1.10μM and 1.66±1.06μM respectively and inhibit IP6K3 with an IC_50_ of 4.93±1.14μM and 11.10±1.13μM respectively. Reactions were run with (A) 1mM ATP, 100μM IP6, 7.5nM IP6K2, for 30 minutes at 37°C or (B) 1mM ATP, 100μM IP6, 120nM IP6K3, for 120 minutes at 37°C. Percent activity is defined as 100*(μ_experimental_-μ_negative_)/(μ_positive_–μ_negative_) where positive is the experimental result with no inhibitor present and negative is the signal produced at the highest concentration of inhibitor.

Myricetin and 6-Hydroxy-DL-Dopa are both promiscuous molecules and were not expected to specifically inhibit IP6K1. In fact, Myricetin is annotated in the LOPAC as being an inhibitor of a number of kinases. We determined the IC_50_ values for each of these compounds against the rest of the IP6K family, IP6K2 and IP6K3. Myricetin was found to inhibit IP6K2 with an IC_50_ value of 23.41±1.10μM (Fig 9A) and IP6K3 with an IC_50_ value of 4.93±1.14μM (Fig 9B). IP6K2 and IP6K3 were also inhibited by 6-Hydroxy-DL-Dopa by IC_50_ = 1.66±1.06μM (Fig 9A) and 11.10±1.13μM (Fig 9B) respectively.

## Conclusions

In the current study we have designed, optimized and validated a high-throughput assay to measure the activity of IP6K1 and discover novel inhibitors. We have confirmed that the assay works with a known IP6K1 inhibitor, TNP, whose mechanism of action is through ATP competition. The assay was optimized to perform well at physiologically relevant ATP concentrations. Screening the LOPAC afforded a hit rate of 1.88% and discovered two previously unknown IP6K1 inhibitors, Myricetin and 6-Hydroxy-DL-Dopa, which were further confirmed with a dose-response analysis, inositol phosphate PAGE separation, and did not significantly inhibit the secondary detection steps. Furthermore, the assay displayed robust characteristics with a Z’ factor of 0.62, a signal to background ratio of 6, and a CV% of 8.5. Taken together these results lend confidence in the ability to detect real hits using this assay. Therefore, the assay described here can serve as a basis for larger high-throughput screening programs for the discovery of novel IP6K1 inhibitors that can be used to study the function of IP6K1 as well as validate the enzyme as a drug target.

## Supporting information

S1 Appendix Effect of IP6 on ADP standard curve. (XLSX)

S2 Appendix Raw signal and Z-score of all compounds screened in the LOPAC. (XLSX)
